# PINK1-dependent phosphorylation of PINK1 and Parkin is essential for mitochondrial quality control

**DOI:** 10.1038/cddis.2016.396

**Published:** 2016-12-01

**Authors:** Na Zhuang, Lin Li, She Chen, Tao Wang

**Affiliations:** 1 School of Life Sciences, Tsinghua University, Beijing, China; 2 National Institute of Biological Sciences, Beijing, China

## Abstract

Mitochondrial dysfunction has been linked to the pathogenesis of a large number of inherited diseases in humans, including Parkinson's disease, the second most common neurodegenerative disorder. The Parkinson's disease genes *pink1* and *parkin*, which encode a mitochondrially targeted protein kinase, and an E3 ubiquitin ligase, respectively, participate in a key mitochondrial quality-control pathway that eliminates damaged mitochondria. In the current study, we established an *in vivo* PINK1/Parkin-induced photoreceptor neuron degeneration model in *Drosophila* with the aim of dissecting the PINK1/Parkin pathway in detail. Using LC-MS/MS analysis, we identified Serine 346 as the sole autophosphorylation site of *Drosophila* PINK1 and found that substitution of Serine 346 to Alanine completely abolished the PINK1 autophosphorylation. Disruption of either PINK1 or Parkin phosphorylation impaired the PINK1/Parkin pathway, and the degeneration phenotype of photoreceptor neurons was obviously alleviated. Phosphorylation of PINK1 is not only required for the PINK1-mediated mitochondrial recruitment of Parkin but also induces its kinase activity toward Parkin. In contrast, phosphorylation of Parkin by PINK1 is dispensable for its translocation but required for its activation. Moreover, substitution with autophosphorylation-deficient PINK1 failed to rescue *pink1* null mutant phenotypes. Taken together, our findings suggest that autophosphorylation of PINK1 is essential for the mitochondrial translocation of Parkin and for subsequent phosphorylation and activation of Parkin.

Mitochondria have a fundamental role in eukaryotic metabolic processes by generating adenosine triphosphate as a cellular energy source. Dysfunctional mitochondria deprive cells of energy, produce toxic reactive oxygen species, and other pro-death mediators to initiate cell death. The mitochondrial quality-control pathways that evolved to maintain the integrity of mitochondria therefore have key roles in the normal function of cells.^1, 2^ The pathogenesis of a large number of inherited diseases in humans, including Parkinson's disease, has been linked to mitochondrial dysfunction.^3, 4, 5^ The *pink1* and *parkin* genes encode a mitochondrially targeted protein kinase and an E3 ubiquitin ligase, respectively. Mutations in both genes are known to cause early-onset Parkinson's disease.^6, 7^ A large number of studies have demonstrated that PINK1 functions upstream of Parkin in a key mitochondrial quality-control pathway that is known as mitophagy.^8, 9^

Mitophagy is a selective form of autophagy that targets dysfunctional mitochondria for lysosomal degradation and protects cells from mitochondrial damage.^10^ In damaged mitochondria, PINK1 accumulates on the outer membrane (OMM) of depolarized mitochondria.^11, 12^ The accumulation of PINK1 is essential both for the recruitment of Parkin onto depolarized mitochondria and for the activation of Parkin. Parkin has been shown to initiate ubiquitination of numerous OMM proteins, to initiate selective autophagy, and to function in the degradation of damaged mitochondria.^8, 13, 14, 15, 16^ PINK1 kinase activity is required to induce the translocation of Parkin to depolarized mitochondria, and several mutations in the *pink1* gene that yield stable PINK1 that lacks kinase activity have been associated with Parkinsonisms.^17, 18, 19^

PINK1 accumulated on the OMM has been shown to phosphorylate Parkin and is required for the recruitment of Parkin.^20, 21, 22, 23, 24, 25^ Studies with mammalian cells have shown that, upon depolarization, PINK1 can autophosphorylate at residues S228 and S402, a situation that also appears to be involved in the recruitment of Parkin.^24, 26^ Recently, it has been suggested that S402 phosphorylation appears to be important for PINK1 function, as this is known to be involved in PINK1 dimerization, Parkin recruitment, and the induction of mitophagy.^24, 27^ However, despite the fact that many PINK1 substrates have been identified, precisely how PINK1 kinase activity is involved in the recruitment of Parkin to the depolarized mitochondria remains unclear.

In this study, we established a PINK1/Parkin-induced cell death model in *Drosophila*. We identified a single PINK1 autophosphorylation site at the S346 residue, which is conserved as S228 in mammals. Expression of a truncated PINK1 with an S346A mutation abolished PINK1-induced phosphorylation of both PINK1 and Parkin, the recruitment of Parkin to mitochondria, and consequent cell death. Moreover, PINK1^S346A^ failed to rescue *pink1* null mutant phenotypes, whereas both PINK1^S519A^ and PINK1^S346D^ fully rescued these phenotypes. These results demonstrate that PINK1-dependent phosophorylation of both PINK1 and Parkin is required for activating PINK1/Parkin signaling and highlight the fact that PINK1 autophosphorylation is a key event for mitochondrial translocation and the activation of Parkin.

## Results

### Establishment of an *in vivo* PINK1/Parkin-induced photoreceptor neuron degeneration model

**Figure 1:**
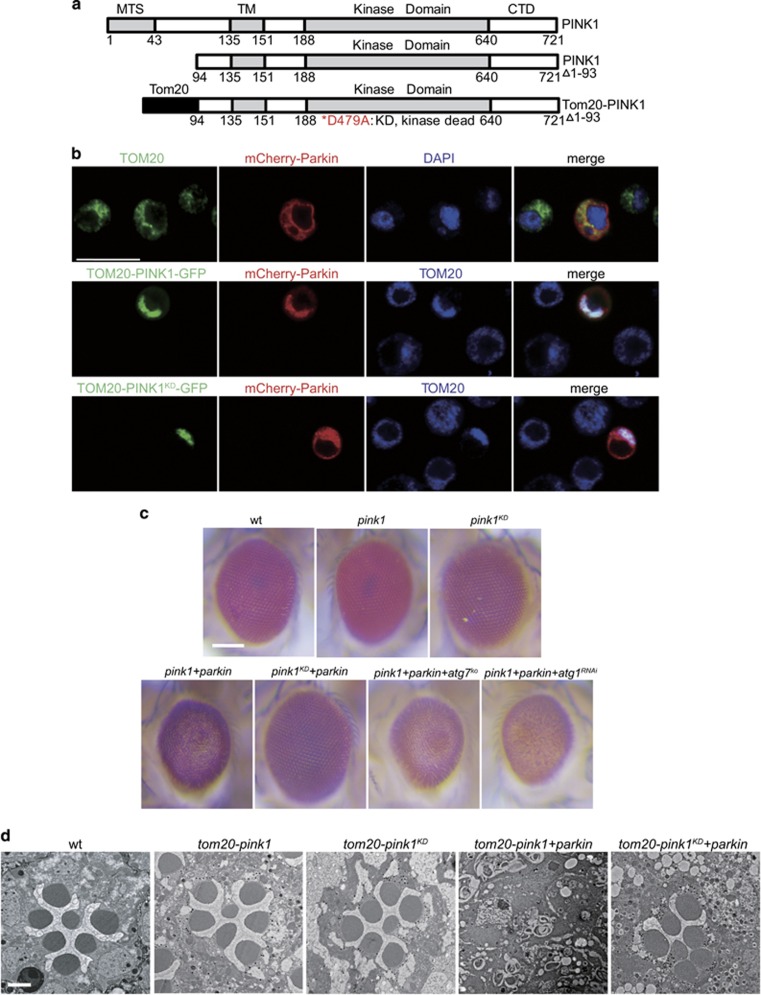
Establishment of an *in vivo* PINK1/Parkin-induced photoreceptor neuron degeneration model. (**a**) Schematic diagram describing the construction of *Drosophila* full-length PINK1, truncated PINK1 (94–721aa), and recombinant TOM20-PINK1-GFP (TOM20:1–50aa+PINK1:94–721aa). (**b**) Confocal images of *Drosophila* S2 cells expressing mCherry-Parkin (red) alone or coexpressing mCherry-Parkin (red) and TOM20-PINK1-GFP (green) or TOM20-PINK1^KD^-GFP (green). Mitochondria are labeled with the anti-TOM20 antibody (upper: green; middle and lower: blue). Scale bar represents 20 *μ*m. (**c** and **d**) Expression of mitochondria OMM stabilized PINK1 and Parkin together in all tissues of the fly eye was sufficient to induce cell degeneration. Stereo fluorescence microscopic images (**c**) and TEM images (**d**) of *Drosophila* compound eyes of wild-type (wt), *GMR-tom20-pink1-GFP* (*pink1*), *GMR-tom20-pink1*^*KD*^*-GFP* (*pink1*^*KD*^), *GMR-tom20-pink1-GFP/GMR-flag-parkin* (*pink1+parkin*), *GMR-tom20-pink1*^*KD*^*-GFP/GMR-flag-parkin* (*pink1*^*KD*^*+parkin*), *atg7*^*d7*^*;GMR-tom20-pink1-GFP/GMR-flag-parkin* (*pink1+parkin+atg7*^*KO*^) or *GMR-gal4/UAS-atg1*^*RNAi*^*;GMR-tom20-pink1-GFP/GMR-flag-parkin* (*pink1+parkin+atg1*^*RNAi*^). Scale bar on panels (**c**) and (**d**) represents 100 and 2 *μ*m, respectively

PINK1 is known to be imported and degraded in mitochondria in normal conditions. However, in HeLa cells, stabilized PINK1 on the OMM recruits Parkin and causes the elimination of mitochondria.^8, 12^ To establish an *in vivo* system of PINK1-induced activation of mitochondrial quality-control pathway, we replaced residues 1–93 of PINK1 with the OMM anchor from the N-terminal region of TOM20, and TOM20-PINK1-GFP stably accumulated on the mitochondrial in normal conditions (Figure 1a). In Schneider 2 (S2) cells, upon coexpression with mCherry-Parkin, which was randomly distributed in cytosol when expressed by itself, TOM20-PINK1-GFP recruited mCherry-Parkin to mitochondria (Figure 1b). In contrast, PINK1 with a kinase-dead (PINK1^D479A^ or PINK1^KD^) mutation prevent the mitochondrial recruitment of Parkin (Figure 1b).

The *Drosophila* compound eye is a powerful system to study the pathogenesis of neurodegenerative diseases.^28^ We next expressed GFP-tagged TOM20-PINK1 proteins in the fly compound eyes using the *GMR* (*Glass Multiple Reporter*) promoter and found that both *GMR-tom20-pink1-GFP* and *GMR-tom20-pink1*^*KD*^*-GFP* flies had truncated PINK1 expressed and normal eye morphology (Figure 1c). To check the cellular effects of expressing truncated PINK1 in detail, we examined the morphology of retinae by transmission electron microscopy (TEM). Each ommatidium from normal compound eyes contains seven intact photoreceptor cells with microvillar structure named rhabdomere. Each individual ommatidium from *GMR-tom20-pink1-GFP* compound eyes contained a full complement of seven intact photoreceptor cells (Figure 1d). We reasoned that the lack of obvious cell death observed in the mitochondrial-targeted PINK1 in photoreceptor neurons might result from the low expression level of endogenous Parkin in compound eyes. Therefore, we additionally expressed Flag-tagged Parkin with the *GMR* promoter. Coexpression of TOM20-PINK1-GFP and Flag-Parkin resulted in glassy eyes with an absence of pigmentation, representing severe degeneration of retina cells, whereas the eye morphology of the *GMR-tom20-pink1*^*KD*^*-GFP*/*GMR-flag-parkin* flies was normal (Figure 1c). These differences did not result from low expression levels of PINK1^KD^, as both *GMR-tom20-pink1*^*KD*^*-GFP* and *GMR-tom20-pink1-GFP* had similar PINK1-GFP protein levels (Supplementary Figure S1). To verify the degeneration phenotype of photoreceptor neurons detected externally, we examined the morphology of retinae by TEM. The *GMR-tom20-pink1-GFP/GMR-flag-parkin* flies completely lacked photoreceptor cells, whereas there was no loss of photoreceptor cells and/or rhabdomeres observed in the *GMR-tom20-pink1*^*KD*^*-GFP/GMR-flag-parkin* flies (Figure 1d).

To investigate whether autophagy was involved in the retinal cell death, we coexpressed TOM20-PINK1-GFP and Flag-Parkin in an autophagy-deficient background. Both *atg1*^*RNAi*^and *atg7*^*KO*^ failed to alleviate the degeneration of photoreceptor cells (Figure 1c). We next assessed whether loss of mitochondria was associated with PINK1/Parkin-induced cell death. First, we checked mitochondrial membrane potential using JC-10 assay. The addition of CCCP resulted in the complete loss of membrane potential, whereas the expression of TOM20-PINK1 and Parkin did not affect mitochondrial membrane potential, indicating that the PINK1/Parkin-induced cell death is irrespective of the mitochondrial membrane potential (Supplementary Figure S2). Furthermore, mitochondrial proteins, including ND42, COXIV, and Acon, did not decrease in the degenerating retina, indicating that PINK1/Parkin-mediated cell death is not caused by mitochondrial loss (Supplementary Figure S3). Therefore, the PINK1/Parkin-induced cell death is independent of mitochondrial autophagy.

### PINK1 is autophosphorylated on Serine 346

**Figure 2:**
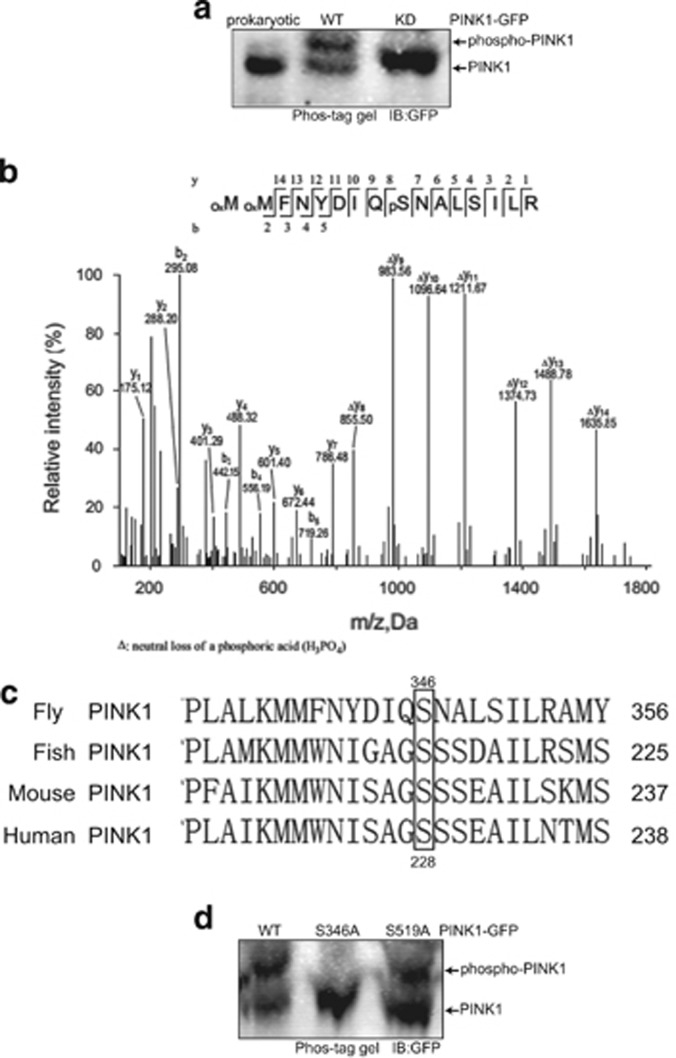
PINK1 is autophosphorylated on Serine 346. (**a**) Whole-cell lysates of *Drosophila* S2 cells transfected with *tom20-pink1-GFP* or *tom20-pink1*^*KD*^*-GFP* were analyzed with Phos-tag gel electrophoresis. Prokaryotic expressed TOM20-PINK1-GFP was used as an internal control for unmodified PINK1 (lane 1). Anti-GFP antibody was used for immunoblotting analysis. (**b**) Tandem mass (MS/MS) spectrum analysis of the *in vivo* autophosphorylation site of *Drosophila* PINK1. TOM20-PINK1-GFP or TOM20-PINK1^KD^-GFP purified from transgenic fly heads of *GMR-tom20-pink1-GFP* or *GMR-tom20-pink1*^*KD*^*-GFP* was subjected to LC-MS/MS analysis. A sole phosphorylated peptide, equivalent to amino acids 338–353, was identified only from *tom20-pink1-GFP* transgenic flies. The b and y ions are marked in the spectrum and also illustrated along the peptide sequence shown on top of the spectrum. The subscript p marks a phosphorylation modification on Ser-346. (**c**) Multiple sequence alignment of PINK1 residues neighboring S346 with various organisms. S346 (boxed) has been evolutionarily conserved across all species. (**d**) Serine 346 was confirmed to be the sole autophosphorylation site of PINK1. Whole-cell lysates of Drosophila S2 cells transfected with wild-type or mutant forms of TOM20-PINK1-GFP were subjected to phos-tag gel electrophoresis. The high molecular shift in the phos-tag gel completely disappeared in the S346A mutation but was unaffected in the S519A mutation

We then asked whether PINK1 is autophosphorylated when stably accumulated on mitochondria. We first used Phos-tag gels to investigate PINK1 phosphorylation. Wild-type PINK1 with TOM20 exhibited a shift on the Phos-tag gel, whereas TOM20-PINK1^KD^ or prokaryotic expressed TOM20-PINK1 did not display a gel shift, suggesting that PINK1 has an autophosphorylation modification (Figure 2a). To identify potential PINK1 autophosphorylation sites, we used electrospray tandem mass spectrometry (LC-MS/MS) analysis, and a single phosphorylation site was identified (Ser346) on TOM20-PINK1-GFP that was not present on TOM20-PINK1^KD^-GFP. Importantly, S346 is a conserved site with S228 that has been identified in mammalian cells^26^ (Figures 2b and c). To verify whether S346 was indeed the autophosphorylation site of PINK1, we exchanged Serine 346 for Alanine. The gel shift band in the phos-tag gel completely disappeared in the S346A mutation, as was the case with PINK1^KD^, suggesting that S346 is the sole autophosphorylation site of PINK1 (Figure 2d). Moreover, we substituted another autophosphorylation site of PINK1 to Alanine that had been identified in previous mammalian studies, S519 (the homologous site of S402 in humans, Supplementary Figure S4), and found that the autophosphorylation signal was not changed in the PINK1^S519A^ mutant (Figure 2d and Supplementary Figure S4). These results indicated that PINK1 autophosphorylation occurs at Serine 346 in *Drosophila*.

### PINK1 phosphorylation is required for the activation of PINK1/Parkin signaling

**Figure 3:**
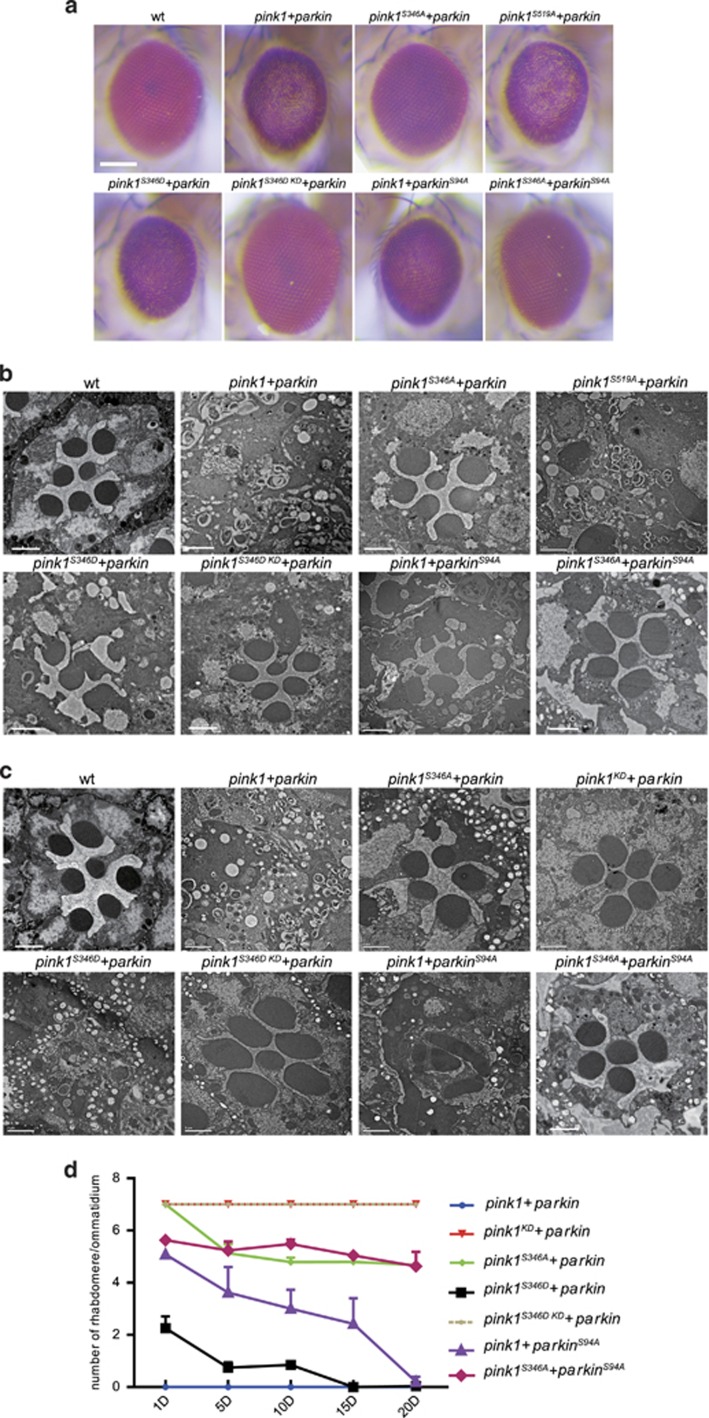
Phosphorylation of both PINK1 and Parkin is required for activation of the PINK1/Parkin pathway. (**a**) Stereo fluorescence microscopic images of *Drosophila* compound eyes from wt, *GMR-tom20-pink1-GFP/GMR-flag-parkin* (*pink1+parkin*), *GMR-tom20-pink1*^*S346A*^*-GFP/GMR-flag-parkin* (*pink1*^*S346A*^*+parkin*), *GMR-tom20-pink1*^*S519A*^*-GFP/GMR-flag-parkin* (*pink1*^*S519A*^*+parkin*), *GMR-tom20-pink1*^*S346D*^*-GFP/GMR-flag-parkin* (*pink1*^*S346D*^*+parkin*), *GMR-tom20-pink1*^*S346D KD*^*-GFP/GMR-flag-parkin* (*pink1*^*S346D KD*^*+ parkin*), *GMR-tom20-pink1-GFP/GMR-flag-parkin*^*S94A*^ (*pink1+parkin*^*S94A*^), and *GMR-tom20-pink1*^*S346A*^*-GFP/GMR-flag-parkin*^*S94A*^ (*pink1*^*S346A*^*+parkin*^*S94A*^). PINK1 with phosphorylation deficient (Serine to Alanine) mutation on residue 346 (S346A) but not on residue 519 (S519A) abolished the photoreceptor neuron degeneration phenotype, whereas phosphorylation mimic (Serine to Aspartic acid) mutation on residue 346 (S346D) of PINK1 caused obvious external eye degeneration, although this was less severe than wild-type PINK1. Scale bar represents 100 *μ*m. (**b** and **c**) TEM images of the single ommatidia from flies that were 1 day old (**b**) and 10 days old (**c**), respectively. Scale bars within the panels represent 2 *μ*m. (**d**) Quantification of the photoreceptor neuron degeneration rate determined by optical neutralization assay. The graph shows the mean with S.D. of the number of rhabdomeres per ommatidium in ⩾3 flies, with ⩾108 ommatidia counted per sample

To investigate the *in vivo* role of PINK1 autophosphorylation, we expressed TOM20-PINK1-GFP with a S346A or a S346D mutation in the fly compound eyes. Unlike wild-type PINK1, coexpression of TOM20-PINK1^S346A^-GFP and Flag-Parkin did not lead to external retinal degeneration on the first day (Figure 3a). However, consistent with the phos-tag gel results, mutation on the other putative phosphorylation site of PINK1 (PINK1^S519A^) caused a severe absence of pigmentation upon coexpression with Parkin as the similar extension as with wild-type PINK1 (Figure 3a). These results emphasize again that S346 is the sole autophosphorylation site of PINK1. Coexpression of the TOM20-PINK1^S346D^-GFP and Flag-Parkin caused external eye degeneration, although this was not as severe as for wild-type PINK1. The different effects of PINK1^S346A^ and PINK1^S346D^ on retinal degeneration are not due to expressing different levels of PINK1 (Supplementary Figure S1). However, coexpression of Parkin reduced the stability of both wild-type PINK1 and PINK1^S346D^, which indicated that PINK1 might also be the substrate of Parkin (Supplementary Figure S5). We used TEM to evaluate photoreceptor neuron integrity. Similar to the light microscopic results, the expression of Parkin with either wild-type TOM20-PINK1 or TOM20-PINK1^S519A^ completely abolished photoreceptor cells in young flies (<1-day old, Figure 3b). A severe loss of photoreceptor cells was also detected in *GMR-pink1*^*S346D*^*-GFP*/*GMR-flag-parkin* flies, but this did not occur in *GMR-pink1*^*S346A*^*-GFP/GMR-flag-par*k flies at day 1 (Figure 3b). To further check whether the unphosphorylated PINK1 caused neurodegeneration in aged animals, we performed optical neutralization assay to count the number of rhabdomeres per ommatidium during aging^29^ (Figure 3d). We found that *GMR-pink1*^*S346D*^*-GFP*/*GMR-flag-parkin* flies displayed a near complete loss of photoreceptor cells on day 10, whereas flies expressing PINK1^S346A^ reserved most of their photoreceptor cells (Figures 3c and d). Therefore, autophosphorylation of PINK1 on S346 is required for the activation of the PINK1/Parkin pathway.

### Phosphorylation of Parkin is required for the activation of the PINK1/Parkin pathway

We next queried whether PINK1 autophosphorylation alone was sufficient for the activation of the PINK1/Parkin pathway. We introduced a S346D substitution into PINK1^KD^ (PINK1^S346D KD^), and the coexpression of TOM20-PINK1^S346D KD^-GFP and Flag-Parkin in the compound eyes did not lead to the death of retinal cells even in aged animals, a result indicating that substrates other than PINK1 are important for the activation of the PINK1/Parkin pathway (Figure 3). As it is well established that Parkin is an important substrate of PINK1 kinase, we tested whether phosphorylation of Parkin was essential for the proper function of PINK1. Ser65 is the major phosphorylation site of human Parkin, and this residue is conserved with *Drosophila* Parkin (Ser94)^21, 22, 30^ (Supplementary Figure S6A). Therefore, we substituted the Ser94 residue with Ala to abolish this PINK1-induced Parkin phosphorylation and expressed Parkin^S94A^ in the compound eyes (Supplementary Figures S6B and C). Coexpression of TOM20-PINK1 and Parkin^S94A^ did not result in a severe retinal degeneration phenotype as with wild-type Parkin (Figures 3a and b), which suggested that PINK1 kinase activity toward Parkin is also essential for the activation of the PINK1/Parkin pathway.

To further check whether *GMR-tom20-pink1/GMR-flag-parkin*^*S94A*^ flies underwent an age-dependent retinal degeneration, we counted the number of rhabdomeres per ommatidium on aged animals (Figure 3d). The flies with TOM20-PINK1 and Parkin^S94A^ coexpressed displayed a mild age-dependent loss of photoreceptor cells (Figures 3c and d). Moreover, *GMR-pink1*^*S346A*^*-GFP*/*GMR-flag-parkin*^*S94A*^ flies displayed a similar severity of photoreceptor cell degeneration as flies with PINK1^S346A^ and wild-type Parkin coexpressed, which indicated that Parkin phosphorylation is an event downstream of autophosphorylation of PINK1 on S346 (Figures 3c and d). The more profound suppressive effect of phosphorylation-deficient substitution on PINK1 over Parkin suggested that PINK1-mediated phosphorylation of PINK1 and Parkin might have different roles in the mitochondrial quality-control pathway. Nevertheless, phosphorylation of both PINK1 and Parkin is required for the activation of PINK1/Parkin signaling.

### Parkin recruitment by PINK1 is dependent on phosphorylation of PINK1 but independent of phosphorylation of Parkin

**Figure 4:**
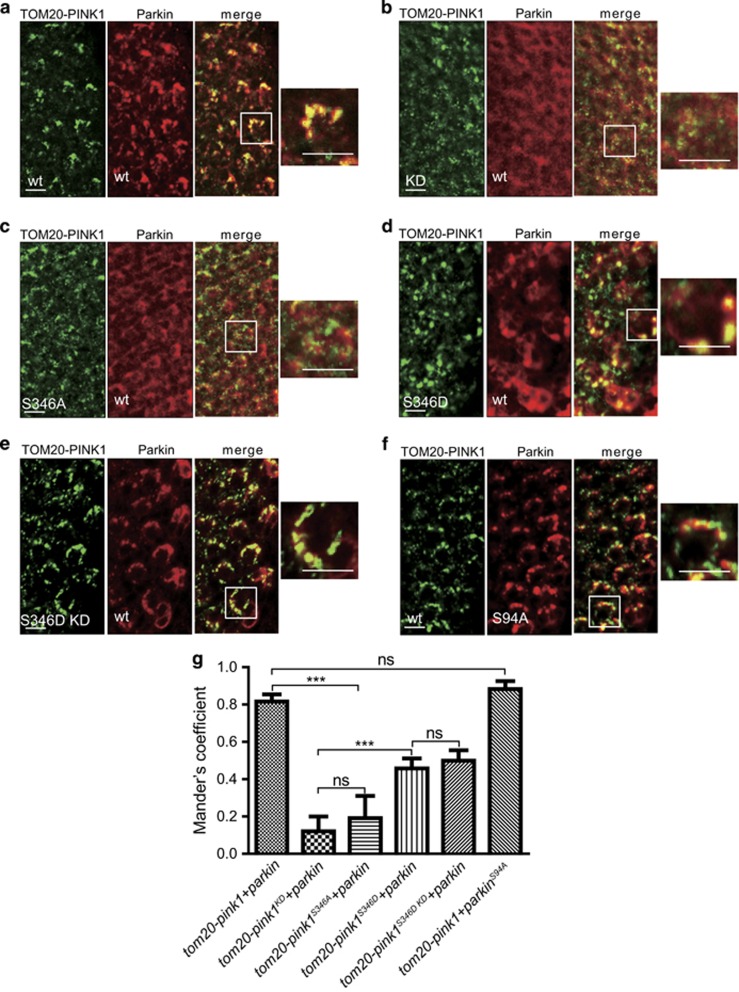
Parkin mitochondrial recruitment is dependent on phosphorylation of PINK1 but independent of phosphorylation of Parkin. (**a**–**f**) Confocal images of *Drosophila* third instar eye discs when ectopic TOM20-PINK1-GFP and Flag-Parkin are starting to be expressed. The GFP (green) fluorescence of TOM20-PINK1 indicates the localization of mitochondria, and Parkin is stained by anti-FLAG (red) antibody. The scale bars within the images represent 5 *μ*m. (**a**) Wild-type PINK1 colocalized with Parkin on mitochondria. Genotype: *GMR-tom20-pink1-GFP/GMR-flag-parkin.* (**b**) PINK1 without kinase activity failed to recruit Parkin. Genotype: *GMR-tom20-pink1*^*KD*^*-GFP/GMR-flag-parkin.* (**c**) PINK1 with a phosphorylation deficient (S346A) mutation did not recruit Parkin efficiently. Genotype: *GMR-tom20-pink1*^*S346A*^*-GFP/GMR-flag-parkin.* (**d** and **e**) A phosphorylation mimic mutation (S346D) of PINK1 induced statistically significant mitochondrial redistribution of Parkin even without kinase activity. Genotype: (**d**) *GMR-tom20-pink1*^*S346D*^*-GFP/GMR-flag-park* and (**e**) *GMR-tom20-pink1*^*S346D KD*^*-GFP/GMR-flag-park.* (**f**) Parkin with an S94A mutation that abolished PINK1-mediated phosphorylation still efficiently colocalized with TOM20-PINK1. Genotype: *GMR-tom20-pink1-GFP/GMR-flag-parkin*^*S94A*^. (**g**) Quantification of the efficiency of mitochondria translocation of Parkin. The Mander's Overlap Coefficient was used to measure colocalization of Parkin and PINK1 in confocal fluorescence microscopic images. The graph shows the mean with S.D. of the Mander's colocalization coefficient between TOM20-PINK1 (green) and flag-Parkin (red) for 3 independent experiments, with at least 10 eye discs counted per sample. Significant differences were determined using Student's *t*-test (NS, not significant; ***P<0.001)

We reasoned that the abolishment of the phosphorylation of PINK1 or Parkin might fail to activate the PINK1/Parkin pathway by disrupting the Parkin recruitment to the OMM and/or causing impairment of Parkin activity. We tested the first hypothesis by examining the localization of Parkin in the third instar eye discs. Although Parkin colocalized with wild-type PINK1 and TOM20 on mitochondria, PINK1 lacking kinase activity failed to recruit Parkin to mitochondria and had low Mander's coefficient values with Parkin, confirming that PINK1 kinase activity is required for mitochondrial translocation of Parkin *in vivo* (Figures 4a, b, g, Supplementary Figures S7A, B, and G). Similar to PINK1^KD^, PINK1 with the S346A replacement did not efficiently recruit Parkin to mitochondria, whereas the S346D mutation induced Parkin translocation and displayed significantly higher Mander's coefficient values compared with PINK1^KD^ (Figures 4c, d, g, Supplementary Figures S7C, D, and G). However, although Parkin^S94A^ did not cause severe retinal cell degeneration in combination with TOM20-PINK1, it still efficiently colocalized with TOM20-PINK1 and mitochondria when coexpressed (Figures 4f, g, Supplementary Figures S7F and G). To completely exclude the influence of Parkin phosphorylation on its translocation to mitochondria, we also checked localization of Parkin in eye discs expressing TOM20-PINK1^S346D KD^-GFP and Flag-Parkin. We found that PINK1^S346D KD^ had the same Mander's coefficient value with Parkin as PINK1^S346D^ did (Figures 4d, e, g,Supplementary Figures S7D, E, and G). These results suggest that PINK1 autophosphorylation is required and sufficient for Parkin translocation and that PINK1-dependent phosphorylation of Parkin is dispensable for its mitochondrial translocation.

### Autophosphorylation of PINK1 activates its kinase activity toward Parkin

**Figure 5:**
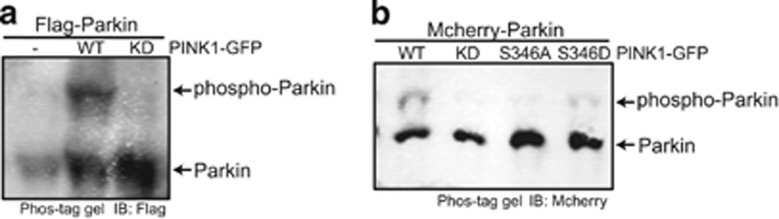
PINK1 autophosphorylation is required for its kinase activity toward Parkin. (**a**) Parkin undergoes PINK1 kinase activity-dependent phosphorylation modification. Whole-cell lysates of S2 cells cotransfected with *flag-parkin* and *tom20-pink1-GFP* or *tom20-pink1*^*KD*^*-GFP* were subjected to phos-tag gel electrophoresis. (**b**) The phosphorylationdeficient PINK1 (S346A) did not phosphorylate Parkin efficiently. S2 cells were cotransfected with *mcherry-parkin* and wild-type or mutant *tom20-pink1-GFP*

As PINK1 autophosphorylation is an upstream event of Parkin phosphorylation, autophosphorylation of PINK1 may be also required for PINK1 kinase activity toward Parkin. To examine this possibility, we coexpressed TOM20-PINK1 and Parkin in S2 cells and checked the phosphorylation status of Parkin. We observed no evidence of Parkin phosphorylation in samples lacking TOM20-PINK1-GFP expression (Figure 5a). However, when coexpressed with TOM20-PINK1, Parkin exhibited a shift that was indicative of phosphorylation (Figure 5a). This phosphorylation modification of Parkin is dependent on PINK1 kinase activity as no phosphorylated band was detected when Parkin was coexpressed with TOM20-PINK1^KD^ (Figure 5a). Moreover, there was no gel shift for Parkin when it was coexpressed with non-phosphorylated PINK1 (PINK1^S346A^) (Figure 5b), which suggested that the phosphorylation on PINK1 S346 activates its kinase activity toward Parkin.

### Autophosphorylation-deficient PINK1 failed to rescue the degeneration phenotypes of the *pink1* mutant flies

**Figure 6:**
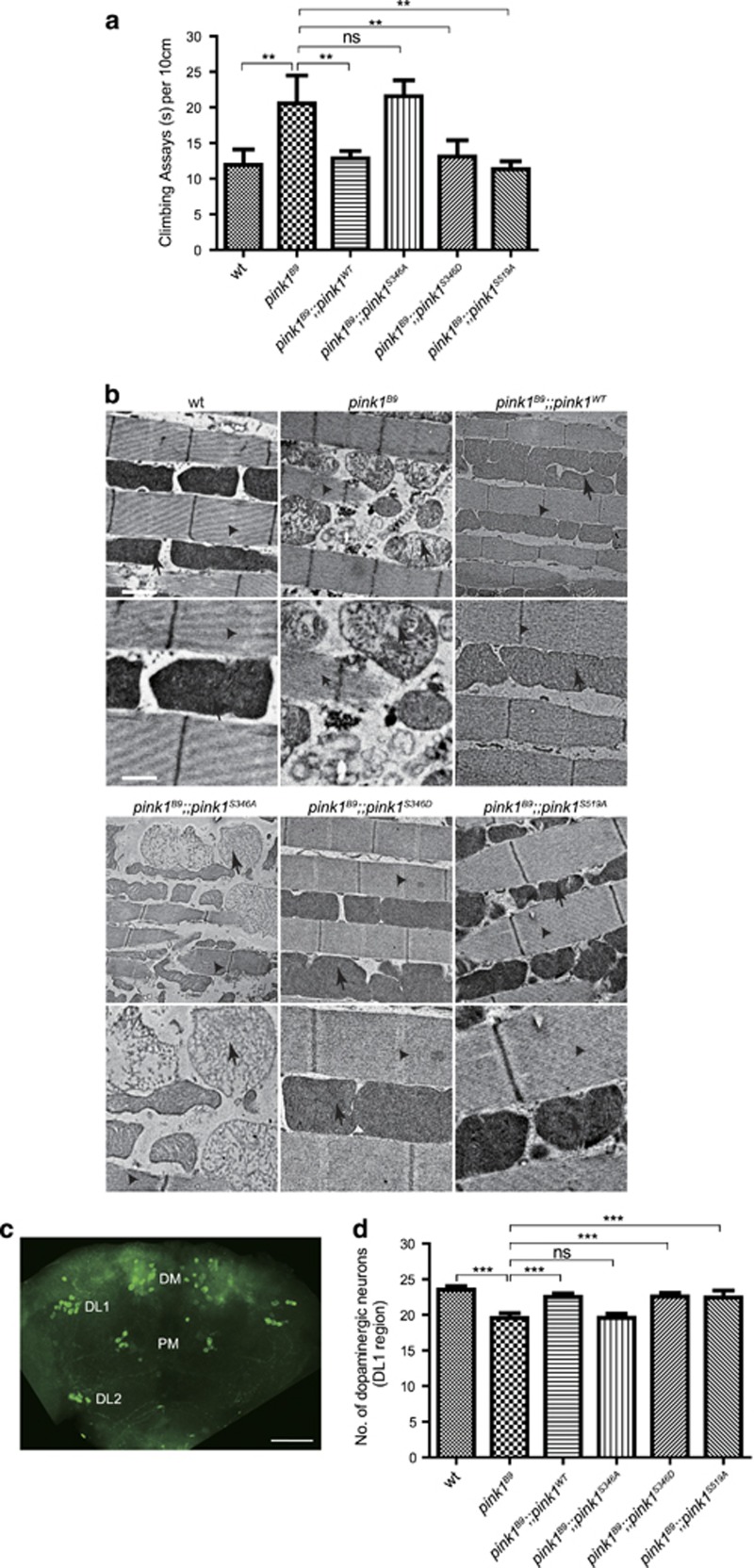
Autophosphorylation-deficient PINK1 failed to rescue the *pink1* mutant phenotypes. (**a**) Expression of the phospho-deficient *pink1*^*S346A*^ transgene failed to rescue abnormal climbing abilities of the *pink1*^*B9*^mutant. Genomic transgenes expressing wild-type PINK1 (*pink1*^*B9*^*;pink1*^*WT*^) or mutant PINK1 (*pink1*^*B9*^*;pink1*^*S346A*^, *pink1*^*B9*^*;pink1*^*S346D*^, and *pink1*^*B9*^*;pink1*^*S519A*^) were introduced into *pink1* null background (*pink1*^*B9*^). Male flies that were 1–3 days old were used for the assay, and significant differences were determined using Student's *t*-test (NS: not significant; **P<0.01; *n*=150–161). (**b**) TEM images of the IFMs from wt, *pink1*^*B9*^, *pink1*^*B9*^*;pink1*^*WT*^, *pink1*^*B9*^*;pink1*^*S346A*^, *pink1*^*B9*^*;pink1*^*S346D*^, and *pink1*^*B9*^*;pink1*^*S519A*^ flies at 3–5 days old. Arrows indicate mitochondria, and arrow heads indicate muscle fibers. The scale bars represent 1 and 0.5 *μ*m on the upper and bottom panels, respectively. (**c** and **d**) Expression of *pink1*^*S519A*^ and *pink1*^*S346D*^ but not *pink1*^*S346A*^ prevented age-dependent loss of dopaminergic neuron in *pink1* mutant brains. (**c**) Whole-mount wild-type adult brains showing dopaminergic neuron clusters marked by anti-TH antibody (green). Scale bar, 50 *μ*m. DM: dorsomedial cluster, DL1: dorsolateral 1 cluster, DL2: dorsolateral 2 cluster, and PM: posteriomedial cluster. (**d**) Quantification of the number of dopaminergic neurons in the DL1 cluster at day 20. At least 15 flies were scored for point. Error bars indicate S.D. Significant differences were determined using Student's *t*-test (NS, not significant; ***P<0.001)

To investigate the *in vivo* roles of PINK1 autophosphorylation, we constructed multiple transgenic lines possessing a *pink1* genomic rescue transgene with different autophosphorylation capacities. These included wild-type PINK1 (*pink1*^*WT*^), phospho-deficient PINK1 (*pink1*^*S346A*^), phospho-mimic PINK1 (*pink1*^*S346D*^), and another putative phospho-deficient PINK1 (*pink1*^*S519A*^). The *pink1*^*B9*^ mutants displayed abnormal wing posture, crushed thorax, and slower climbing speed phenotypes, which were fully rescued by the expression of the wild-type or S346D form of PINK1 (Figure 6a).^31, 32^ However, autophosphorylation-deficient PINK1 (*pink1*^*S346A*^) failed to alleviate the climbing defects of the *pink1*^*B9*^ mutants (Figure 6a). Moreover, consistent with previous results that S346 is the sole autophosphorylation site of *Drosophila* PINK1, *pink1*^*S519A*^ still fully rescued the climbing defects caused by PINK1 deficiency (Figure 6a).

The defective mitochondria in indirect flight muscles (IFMs) is the reason for the downturned wing and reduction of climbing ability in the *pink1*^*B9*^ flies.^31, 32^ We thereby used TEM to examine the effects of PINK1 autophosphorylation on IFMs' mitochondria in detail. Exactly similar to wild-type PINK1, the expression of either *pink1*^*S346D*^or *pink1*^*S519A*^ in the *pink1*^*B9*^ mutant showed normal mitochondrial morphology and densely packed cristae structure in the fly thorax (Figure 6b). In contrast, disorganized muscle fibers and swelled mitochondria with very few cristae were still observed in the *pink1*^*B9*^ mutant, in which a phospho-deficient form of PINK1 (PINK1^S346A^) was expressed (Figure 6b).

The loss of dopaminergic neurons is one of the major characteristics of Parkinson's disease.^33^ We therefore examined whether the autophospho-deficient *pink1* mutants could rescue the dopaminergic neurodegeneration phenotype of *pink1* null mutant. Using immunofluorescent assay with tyrosine hydroxylase (TH) antibody, the number of dopaminergic neurons in dorsolateral (DL1) clusters was counted (Figure 6c).^32^ There was no statistical significance in the number of dopaminergic neurons between wild-type, *pink1*^*B9*^ and *pink1*^*B9*^ with *pink1*^*WT*^, *pink1*^*S346A*^, *pink1*^*S346D*^, and *pink1*^*S519A*^ transgenes expressed on the first day of eclosion. However, at 20 days of age, a small but significant loss of dopaminergic neurons was detected in the *pink1*^*B9*^ flies compared with the wild type (Figure 6d and Supplementary Figure S8). This gradual loss of dopaminergic neurons in the *pink1* mutant animals was fully rescued by the expression of *pink1*^*S346D*^ and *pink1*^*S519A*^ as well as of *pink1*^*WT*^, whereas *pink1*^*S346A*^ did not affect the reduction of dopaminergic neurons in the *pink1*^*B9*^ brain (Figure 6d and Supplementary Figure S8). Taken together, these phenotypic and behavioral analyses strongly support that autophosphorylation of PINK1 on Serine 346 has a key role in its function in mitochondrial quality control *in vivo*.

## Discussion

PINK1 has been reported to phosphorylate many mitochondria-associated proteins, including Mfn2, NdufA10, Bcl-xL, and motor/adaptor complex Miro.^34, 35, 36, 37^ However, phosphorylation of these mitochondrial proteins may not have a key role in PINK1/Parkin-dependent mitophagy, as targeting of PINK1 to other organelles such as peroxisomes and lysosomes that lack these PINK1 substrates can also induce organelle selective autophagy.^12^ Therefore, PINK1 and additional cytosolic targets may be mediators of PINK1 kinase-induced Parkin translocation and mitophagy. We established a genetic model in *Drosophila* of the induction of the PINK1/Parkin pathway through coexpression of mitochondria-targeted PINK1 and Parkin and found that PINK1 and Parkin were both phosphorylated by PINK1 kinase. Furthermore, disruption of PINK1 phosphorylation prevented the mitochondrial translocation of Parkin, and the absence of Parkin phosphorylation abolished PINK1/Parkin-induced cell death. Therefore, PINK1-dependent phosphorylation of both PINK1 and Parkin is essential for the PINK1/Parkin-mediated mitochondrial quality-control pathway.

Mitochondrial uncoupler CCCP depolarizes the mitochondria and induces the selective removal of dysfunctional mitochondria in a PINK1- and Parkin-dependent manner.^38^ However, our study indicates that activated PINK1 and Parkin caused photoreceptor neuron death irrespective of the mitochondrial dysfunction, as coexpression of PINK1 and Parkin did not affect mitochondrial membrane potential. Moreover, activated PINK1- and Parkin-mediated cell death was unaffected by knocking out *atg7* or knocking down *atg1*.^39, 40^ Furthermore, mitochondrial resident proteins remain unchanged in dead retinal cells, indicating that the PINK1/Parkin-induced cell death is not due to a complete loss of mitochondria. In conclusion, consistent with the previous study, PINK1/Parkin induced cell death independently of mitochondrial autophagy.^41^ The molecular mechanisms of the PINK1/Parkin-induced cell death need to be elucidated in future studies.

A previous study reported autophosphorylation of PINK1 observed at residues S228 and S402 following mitochondria depolarization in human cells.^26^ Here we demonstrated that fly PINK1 was autophosphorylated at only one site, Ser346, which is conserved with Ser228 in human PINK1. We showed that substitution of Ser346 with Ala completely abolished PINK1 autophosphorylation. Phosphorylation of human PINK1 on Ser402 has been reported to be involved in PINK1 dimerization, Parkin recruitment, and the induction of mitophagy.^27, 42^ However, we found no evidence of phosphorylation modification at this site in flies. Moreover, *pink1*^*S519A*^ mutants can rescue all the degeneration phenotypes of *pink1*^*B9*^ to the same extent as the wild-type PINK1 did. In contrast, the expression of PINK1^S346A^ failed to rescue the degeneration phenotypes of *pink1* null mutants. Therefore, autophosphorylation of PINK1 on Ser346 is essential for its function in mitochondrial quality control *in vivo*.

PINK1 phosphorylates human Parkin at Ser65, a modification that appears to regulate ubiquitin-ligase activity and mitophagy in cultured cells and in flies.^22, 30, 43^ It has also been reported that Ser65 phosphorylation of human Parkin by PINK1 is required for mitochondrial translocation of Parkin in cultured cells.^21, 44, 45^ However, using a *Drosophila* system, we found that, although the absence of PINK1-mediated phosphorylation of Parkin blocked PINK1/Parkin-induced cell death, phosphorylation-deficient Parkin still translocated to PINK1-targeted mitochondria. This might be because the absence of PINK1-mediated phosphorylation of Parkin blocked its activation without affecting its translocation. Moreover, disruption of PINK1 kinase activity in the autophospho-mimic form of PINK1 did not affect its ability to recruit Parkin. It is clear that phosphorylation of Parkin by PINK1 is not required for Parkin translocation to PINK1-targeted mitochondria.

The autophosphorylation of PINK1 is required but not sufficient to induce Parkin function, as the phospho-mimetic S346D mutants did not activate Parkin and drive cell death when PINK1 lacked its kinase activity. Rather, the autophosphorylation of PINK1 was a prerequisite for its kinase activity toward Parkin, as PINK1 with the unphosphorylated S346A mutation had reduced kinase activity against Parkin. Moreover, autophosphorylation of PINK1 is essential to induce Parkin translocation. However, although autophospho-mimic PINK1 was able to induce Parkin translocation in the absence of kinase activity, the efficiency is not as high as wild-type PINK1. Recent evidence suggests that PINK1 controls translocation and activity of Parkin by phosphorylating ubiquitin.^23, 24, 25, 46^ Therefore, it is possible that PINK1-dependent phosphorylation of ubiquitin also contribute to Parkin mitochondrial translocation.

**Figure 7:**
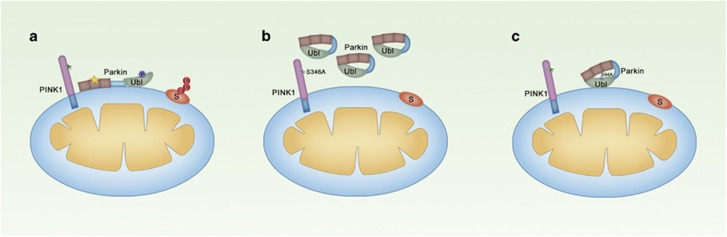
A model of PINK1-mediated phosphorylation in the PINK1/Parkin pathway. (**a**) In damaged mitochondria, PINK1 stabilized on the mitochondrial OMM undergoes autophosphorylation on S346 in normal conditions. The phosphorylated PINK1 promotes Parkin mitochondrial translocation and phosphorylate Parkin on residue S94, which activates Parkin E3 ubiquitin ligase activity to ubiquitinate its mitochondrial substrates for initiating the autophagy of mitochondria. (**b**) Without autophosphorylation modification on S346A mutant, PINK1 fails to recruit, phosphorylate, and activate Parkin. Therefore, inactive Parkin randomly distributes in the cytosol upon phosphorylation deficient PINK1^S346A^ anchored on the mitochondrial OMM. (**c**) Parkin with PINK1-dependent phosphorylation deficient in its ubiquitin-like domain can still be recruited to mitochondria by PINK1 but is not able to activate the PINK1/Parkin pathway

Based on the data from our current work and along with previous work, we propose that PINK1 is autophosphorylated when stabilized on depolarized mitochondria, a process that promotes the mitochondrial translocation of Parkin and increases its kinase activity on Parkin (Figure 7). After being recruited to the mitochondria, Parkin is phosphorylated and activated by PINK1 to ubiquitinate its substrates, further promoting mitophagy (Figure 7). Recent evidence suggests that PINK1 controls Parkin E3 ligase activity not only by phosphorylating Parkin but also by phosphorylating ubiquitin,^23, 24, 25^ and the phosphorylation of ubiquitin on residue Ser65 is also required for Parkin accumulation on damaged mitochondria.^46^ Therefore, it is also possible that phosphorylation of other PINK1 substrates such as ubiquitin is affected by PINK1 autophosphorylation, and such events may have key roles in the PINK1/Parkin pathway.

## Materials and methods

### Drosophila stocks

The fly stocks used were as follows: Transgenic lines, including *GMR-tom20-pink1-GFP, GMR-tom20-pink1^KD^-GFP, GMR-tom20-pink1^S346A^-GFP, GMR-tom20-pink1^S346D^-GFP, GMR-tom20-pink1^S519A^-GFP, GMR-tom20-pink1^S346D KD^-GFP, GMR-flag-parkin, GMR-flag-parkin^S94A^, pink1^WT^, pink1^S346A^,* and *pink1^S346D^*, were generated by site-directed recombination. The *pink1^B9^* is genetic null for *pink1*,^32^ and *atg7^d7^* is null allele for *atg7*.^47^ All flies were maintained at 25 °C.

### Generation of constructs and transgenic flies

The *parkin* cDNA from EST Clone (SD01679) was used to generate the *pIB-mcherry-parkin* and *pIB-flag-parkin* constructs. The *pink1* cDNA was obtained by reverse transcription PCR of total RNA and was then subcloned into the *pMTa-GFP* vector (Invitrogen, Carlsbad, CA, USA) to generate the *tom20-pink1-GFP* construct. The *pink1*^*D479A*^ (*pink1*^*KD*^), *pink1*^*D479A S346D*^ (*pink1*^*S346D KD*^), *pink1*^S346A/D^, *pink1*^*S519A*^, and *pink1*^*S346A S519A*^ mutant constructs were generated via site-directed mutagenesis from the *pMTa-tom20-pink1-GFP* construct. The *parkin*^*S94A*^ mutation was generated via site-directed mutagenesis from the *PIB-flag-parkin* construct.

The wild-type and truncated *pink1* or *parkin* DNA sequences were subcloned into the *pGMR-attB* vector for use in generating the transgenic fly strains. The *pink1* genomic rescue constructs *pink1*^*WT*^, *pink1*^*S346A*^, *pink1*^*S519A*^, and *pink1*^*S346D*^ were generated by replacing the *GMR* sequence of *pGMR-attB-pink1*, *pGMR-attB-pink1*^*S346A*^, and *pGMR-attB-pink1*^*S346D*^ plasmids with *pink1* endogenous promoter sequence (−1291 to +715 base pairs 5′ to the transcription starting site).^31^ The constructs were injected into *M(3xP3-RFP.attPZH-86Fb)* or *M(3xP3-RFP.attPZH-36B)* embryos, and site-specific transformants were identified on the basis of eye color.^48^ All transgenic lines were subsequently tested for protein expression levels by western blotting.

### Cell culture and transfection

*Drosophila* S2 cells were cultured at 28 °C without CO_2_ in Schneider's *Drosophila* Medium (Sigma, St. Louis, MO, USA) containing 10% heat-inactivated fetal bovine serum and 1% Penicillin Streptomycin (Life Technologies, Carlsbad, CA, USA). For cell transfection, S2 cells were transfected with VigoFect transfection reagent (VigoFect, Beijing, China). The transfected cells were then incubated for 24–48 h at 28 °C before analysis.

### Generation of TOM20 antibodies

Polyclonal antibodies against *Drosophila* TOM20 were generated by immunizing a rat with peptides conjugated to KLH. The peptides used to generate the anti-TOM20 antibodies comprised amino acids 128–147 (QEFGNRAAEGNDGPIVLGQS) of *Drosophila* TOM20.

### Transmission electron microscopy

Fly heads or thoraxes were dissected, fixed, dehydrated, and embedded in LR White resin as described previously.^49^ Thin sections (85 nm) were cut with a microtome and examined by TEM (JEM-1400 electron microscope; JEOL, Tokyo, Japan). The images were acquired using a model 832 Gatan camera (Gatan, Inc., Pleasanton, CA, USA).

### Optical neutralization assay

The rhabdomeres of ommatidium were examined directly by the technique of optical neutralization.^29^ Briefly, the heads of flies were immerged into mineral oil in an orientation with antennas facing up and were examined by a DIC light microscope (Nikon, Tokyo, Japan).

### Immunoprecipitation and mass spectrometric analysis

Fly heads were homogenized in buffer A with 1% NP-40. The supernants were then immunoprecipitated using GFP-Trap magnetic beads (Chromotek, Planegg-Martinsried, Germany). After washing twice with buffer A, the beads were resuspended in SDS sample buffer and fractionated by SDS-PAGE. Protein bands on the SDS-PAGE gel excised and de-stained, followed by in-gel digestion with trypsin (10 ng/*μ*l trypsin, 50 mM ammonium bicarbonate, pH 8.0) overnight at 37 °C. Peptides were extracted sequentially with 5% formic acid/50% acetonitrile and 0.1% formic acid/75% acetonitrile and then concentrated by nitrogen purging. The extracted peptides were separated on an analytical capillary column (50 *μ*m × 15 cm) packed with 5 *μ*m spherical C18 reversed phase material (YMC). A Waters nanoAcquity UPLC system (Waters, Milford, MA, USA) was used to generate the following UPLC gradient: 0–30% B in 40 min, 30–70% B in 15 min (A=0.1% formic acid in water, B=0.1% formic acid in acetonitrile). The eluted peptides were introduced via a nano-ESI ion source into an LTQ Orbitrap Velos mass spectrometer (ThermoFisher Scientific, Waltham, MA, USA). The mass spectrometer was operated in data-dependent mode, with one MS scan followed by four CID (collision-induced dissociation) and four HCD (high-energy collisional dissociation) MS/MS scans for each duty cycle. Database searches were performed on an in-house Mascot server (Matrix Science Ltd., London, UK) against the PINK1 protein sequence. Searches examined the following modifications: oxidation on methionine, carbamidomethylation on cysteine, and phosphorylation on serine/threonine/tyrosine. The tandem mass spectra of matched phosphorylated peptides were checked for the phosphorylation site.

### Immunoblotting and phos-tag assay

Fly tissues were homogenized in SDS sample buffer with a pellet pestle (Kimble Chase, Vineland, NJ, USA), and the proteins were fractionated by SDS-PAGE. Proteins from the gels were then transferred onto Immobilon-FL transfer membranes (Millipore, Danvers, MA, USA) in Tris-glycine buffer. The blots were probed with anti-FLAG (Mouse; Sigma, 1:2000 dilution), anti-tubulin (mouse; Developmental Studies Hybridoma Bank, Iowa City, IA, USA, 1:5000 dilution), or anti-GFP (rabbit; Torrey pines Biolabs Inc., San Diego, CA, USA, 1:2000 dilution) as primary antibodies, followed by IRDye 800 goat anti-rabbit IgG (LI-COR) or IRDye 680 goat anti-mouse IgG as secondary antibodies. Signals were detected using an Odyssey infrared imaging system (LI-COR, Lincoln, NE, USA).

To evaluate the phosphorylation levels of the PINK1 and Parkin proteins in the absence of phosphorylation site-specific antibody, 6% polyacrylamide gels containing 50 *μ*M phostag acrylamide (Wako Chemicals, Osaka, Japan) and 100 *μ*M MnCl_2_ were used. After electrophoresis, phostag acrylamide gels were washed in general transfer buffer containing 0.02% SDS and 2 mM EDTA and then replaced with general transfer buffer lacking EDTA. After completely washing out manganese ions, the proteins were transferred onto Immobilon-FL transfer membranes (Millipore) and immunoblotted using a standard protocol.

### Immunostaining

Eye discs from third instar larvae were dissected in PBS solution (pH 6.8) and fixed in 4% paraformaldehyde in PBS buffer for 40 min. The fixed eye discs were incubated in diluted primary anti-serum, rat anti-TOM20 (1:200), mouse anti-Flag (1:500, Sigma), and Rabbit anti-GFP (1:1,000, Invitrogen), followed by staining with secondary antibody anti-Rat Alexa Fluor 647 (1:500, Invitrogen), anti-mouse Alexa Fluor 568 (1:500, invitrogen), and anti-Rabbit Alexa Fluor 488 (1:500, Invitrogen). To detect the colocalization of PINK1, Parkin, and mitochondria in S2 cells, S2 cells were cotransfected with *pMTa-tom20-pink1-GFP* and *PIB-mcherry-parkin*. The transfected cells were fixed with 4% paraformaldehyde and stained with the TOM20 antibody (1:200), followed by anti-Rat Alexa Fluor 647-conjugated secondary antibody (1:500; Invitrogen). Images were captured with an Eclipse Ni microscope confocal microscope (Nikon). The Mander's Overlap Coefficient as a measure of colocalization between Parkin and PINK1 was calculated in confocal fluorescence microscopic images using the ImageJ software (National Institutes of Health, Bethesda, MD, USA). For counting dopaminergic neurons, adult brains were dissected and stained with rabbit anti-TH antibody (1:200, Millipore), followed by anti-Rabbit Alexa Fluor 488 antibody (1:500, Invitrogen). TH immunoreactive cells were imaged with an Eclipse Ni microscope confocal microscope (Nikon), and DA neurons of the DL1 cluster were counted.^50^

### Measurement of mitochondrial membrane potential

The dye JC-10 (Enzo Life Sciences, Farmingdale, NY, USA) was used to evaluate mitochondrial membrane potential. The third instar larval eye discs were dissected and labeled with JC-10 as described.^49^ Images were obtained on a Nikon A1 confocal microscope. The ratio of fluorescence emissions at 525 and 590 nm was used for quantification analysis.
