# Supplementary material for: PINK1-dependent phosphorylation of PINK1 and Parkin is essential for mitochondrial quality control
Source: Cell Death Dis. 2016 Dec 1;7(12):e2501–. doi: 10.1038/cddis.2016.396 (PMC5261015; doi:10.1038/cddis.2016.396)
Supplement: Supplementary Information [file cddis2016396x1.docx]

**Fig.S1. All *tom20-pink1-GFP* transgenic flies had similar PINK1 protein levels.(A-B)**Western blot results revealed the same protein levels of TOM20-PINK1-GFPin the four transgenic lines used in this study: *GMR-tom20-pink1-GFP* (*tom20-pink1*), *GMR-tom20-pink1^KD^-GFP* (*tom20-pink1^KD^*), *GMR-tom20-pink1^S346A^-GFP* (*tom20-pink1^S346A^*), and *GMR-tom20-pink1^S346D^-GFP* (*tom20-pink1^S346D^*). (A) The blot was probed with anti-GFP and anti-tubulin antibodies. (B) Quantification of the relative PINK1-GFP levels. Significant differences were determined using the Student’st-test (ns, not significant).

**Fig. S2. Mitochondrial membrane potential was preserved upon overexpressing PINK1 and Parkin.** **(A)**The Representative JC-10 staining of eye discs. JC-10 fluorescence emissions were captured at 525 nm (green) and 590 nm (red). Eye discs from third instar larva of wild-type,*GMR-tom20-pink1-GFP/GMR-flag-parkin* (*pink1+parkin*), and*GMR-tom20-pink1^KD^-GFP/GMR-flag-parkin* (*pink1^KD^+parkin*)were dissected and treated with or without 10 µM CCCP for 2h. The scale bar within the images represents 100 µm.**(B)** The 525/590 ratio was quantified to indicate the membrane potential.Significant differences were determined with the Student’st-test(ns, not significant; *** p < 0.001).

**Fig. S3.PINK1 and Parkin do not reduce mitochondria contents. (A)**Western blot results revealed that expressing PINK1 and Parkin in compound eyes did not reduce the mitochondrial proteins. Retina from newly eclosed flies expressing TOM20-PINK1 or/and Parkin were dissected. The blot was probed with anti-ND42, anti-COXIV, anti-Acon, and anti-tubulin antibodies.**(B-D)**Quantification of the mitochondria protein levels.Average contents of mitochondrial proteins including ND42, COXIV, and Aconwere normalized to tubulin. Significant differences were determined using the Student’st-test(ns, not significant; * p < 0.05).

**Fig. S4.Multiple sequence alignment revealed that S519 in fly PINK1 is the homologous site of S402 in humanPINK1.**

**Fig. S5. Expression of Parkin reduced the stabilityof PINK1. (A)**Western blot results revealed that the protein levels of wild-typePINK(TOM20-PINK1-GFP) and phosphorylation­mimicPINK1(TOM20-PINK1^S346D^-GFP) decreased upon expression of Parkin. Retina tissues from newly eclosed flies coexpressing PINK1 and Parkinwere dissected, and the blot was probed with anti-GFP, anti-Flag, and anti-tubulin antibodies.**(B)** Quantification of the relative PINK1 and Parkin protein levels.

**Fig. S6.Characterizing the putative PINK1-dependent phosophorylation site on Parkin.(A)**Multiple sequence alignment revealed that S94 in fly Parkin is the homologous site of S65 in humanParkin. **(B-C)**Western blotresults showing the same Parkin protein levels in *GMR-flag-parkin (parkin)* and *GMR-flag-parkin^S94A^(parkin^S94A^)* flies. (B) The blot was probed with anti-flag and anti-tubulin antibodies. (C) Quantification of the relative Flag-Parkin levels. Significant differences were determined using the Student’st-test (ns, not significant).

**Fig. S7.Colocalization of Parkinand mitochondria is dependent on phosphorylation of PINK1, but independent of phosphorylation of Parkin.(A-F)**Thethird instar eye discs were dissected and immunestainedfor Parkinby anti-FLAG (Green) antibody and for mitochondria by anti-Tom20 (Red) antibody.The scale bar within the images represents 5 µm. (A) Parkincolocalized with mitochondria in wild-type PINK1 background. Genotype: *GMR-tom20-pink1-GFP/GMR-flag-parkin.* (B) PINK1 without kinase activity failed to recruit Parkin. Genotype: *GMR-tom20-pink1^KD^-GFP/GMR-flag-parkin.* (C) PINK1 with a phosphorylation­deficient(S346A) mutation did not recruit Parkinefficiently. Genotype: *GMR-tom20-pink1^S346A^-GFP/GMR-flag-parkin.* (D, E) A phosphorylation­mimic mutation (S346D) of PINK1 induced mitochondrial redistribution of Parkin even without kinase activity. Genotype: (D) *GMR-tom20-pink1^S346D^-GFP/GMR-flag-park* and (E) *GMR-tom20-pink1^S346D KD^-GFP/GMR-flag-park.* (F)Parkin with an S94A mutation that abolished PINK1-mediated phosphorylation still efficiently colocalized with TOM20. Genotype: *GMR-tom20-pink1-GFP/GMR-flag-parkin^S94A^.* **(G)**Quantification of the efficiencyof mitochondrial localization of Parkin.The Mander’s Overlap Coefficient was used to measure colocalizationof Parkin and TOM20in confocalfluorescence microscope images. The graph shows the mean with SD of the Mander’scolocalization coefficientbetween TOM20(Red) and Parkin (Green) for three independentexperiments, with at least 10 eye discs counted per sample.

**Fig. S8.Expression of the autophosphorylation-deficient PINK1 failed to alleviate the loss of dopaminergic neurons in *pink1* mutant brains.** Dopaminergic neuron clusters of adult brains were marked by anti-TH antibody (green). The wt, *pink1^B9^*, *pink1^B9^;;pink1^WT^*, *pink1^B9^;;pink1^S346A^*, *pink1^B9^;;pink1^S346D^* and *pink1^B9^;;pink1^S519A^* flies at 20 days old were used. Scale bar, 50 µm.DM: dorsomedial cluster, DL1: dorsolateral 1cluster, DL2: dorsolateral 2 cluster, and PM:posteriomedial cluster.
