# Supplementary figures and images for: PINK1-dependent phosphorylation of PINK1 and Parkin is essential for mitochondrial quality control
Source: Cell Death Dis. 2016 Dec 1;7(12):e2501–. doi: 10.1038/cddis.2016.396 (PMC5261015; doi:10.1038/cddis.2016.396)

Figure S1

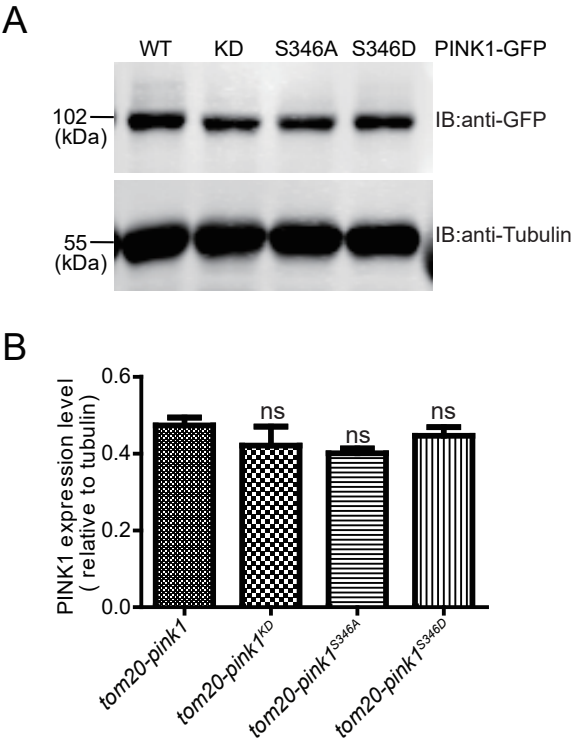

Supplement: Supplementary Figure S1 [file cddis2016396x2.pdf]

Figure S2

A

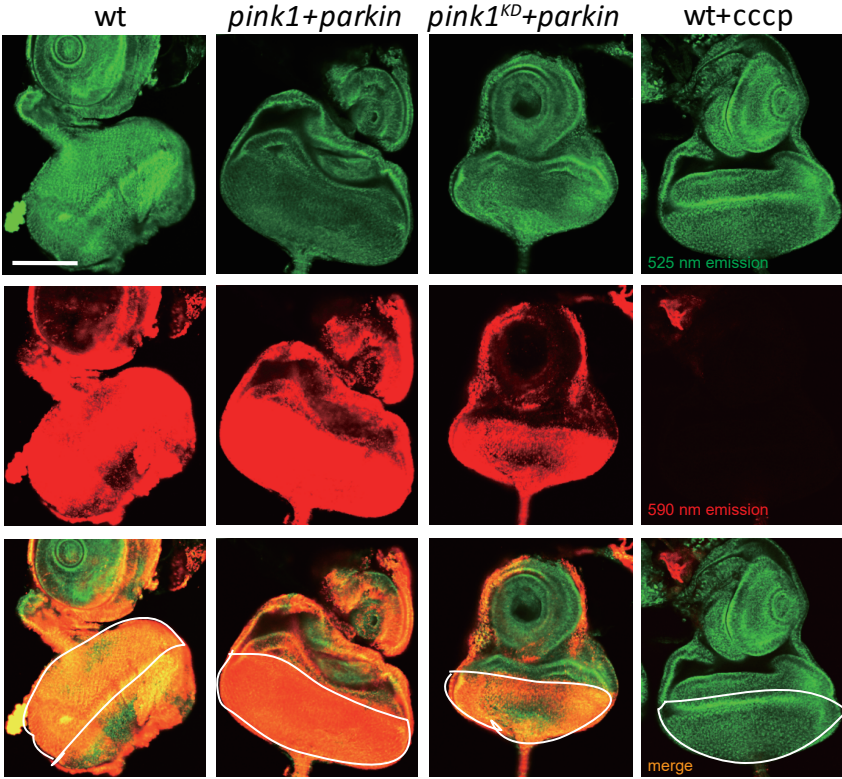

B

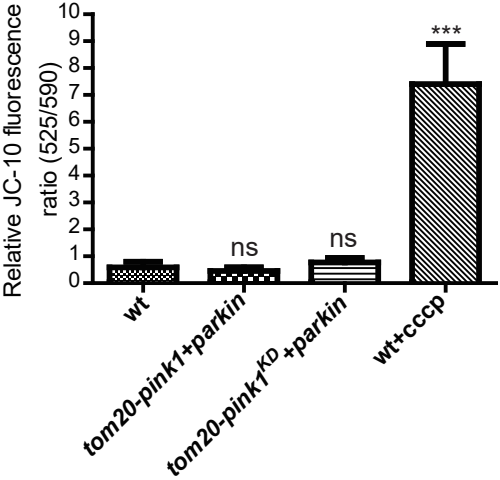

Supplement: Supplementary Figure S2 [file cddis2016396x3.pdf]

Figure S3

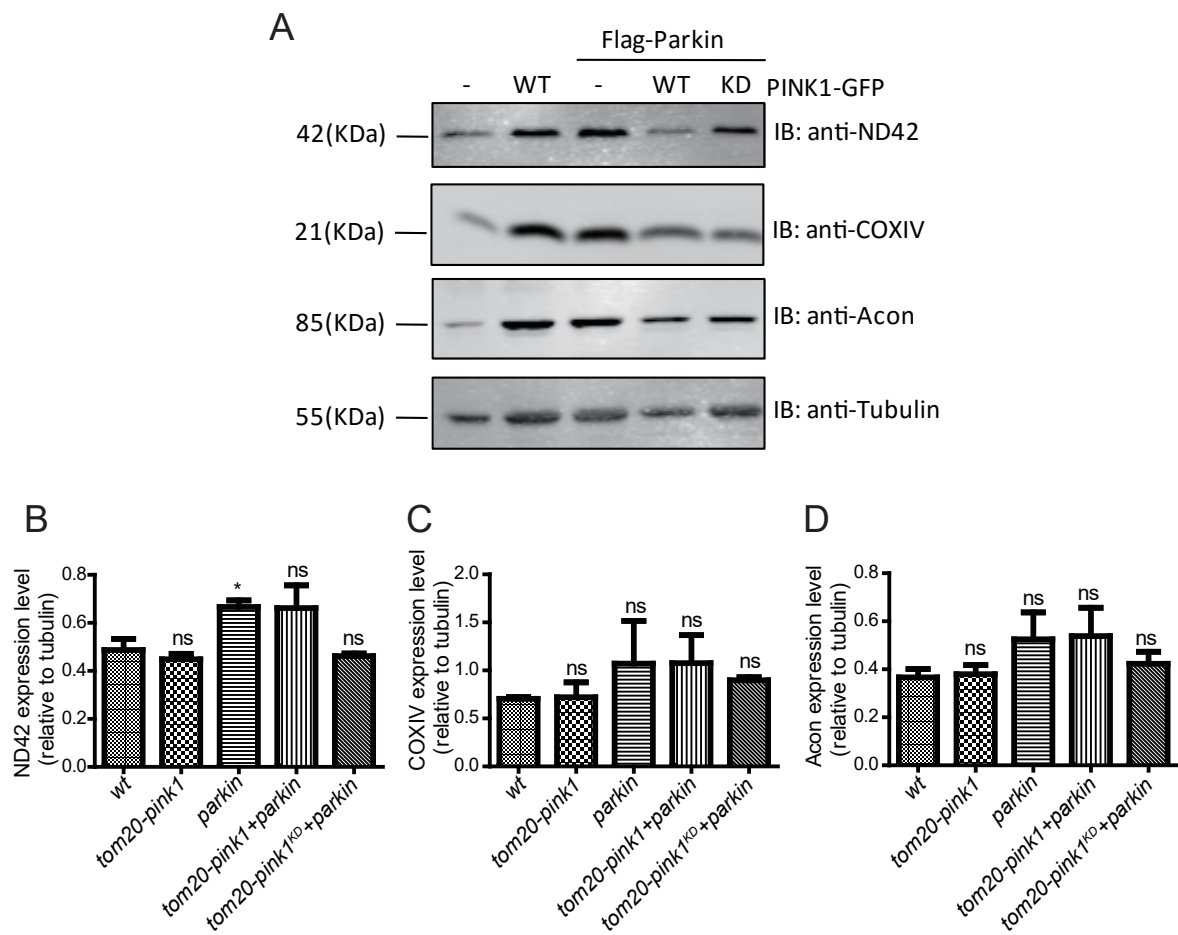

Supplement: Supplementary Figure S3 [file cddis2016396x4.pdf]

Figure S5

A

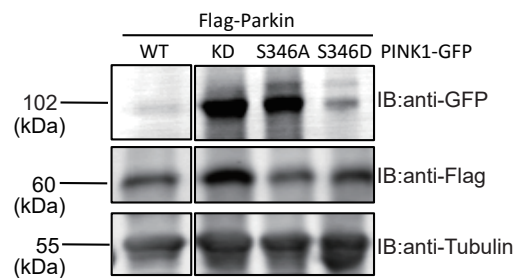

B

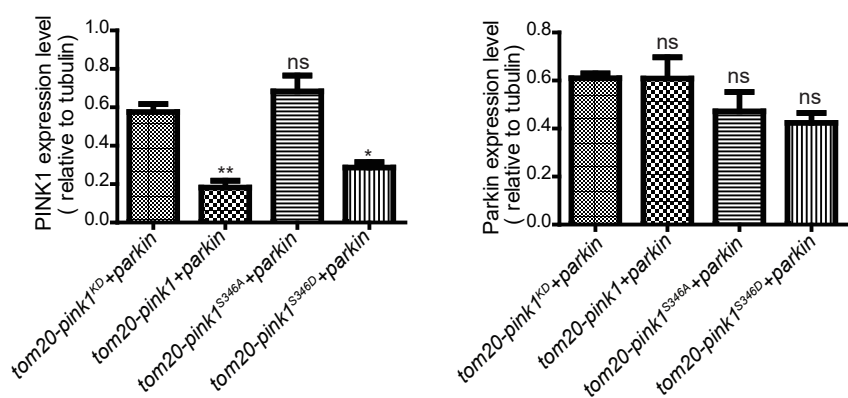

Supplement: Supplementary Figure S5 [file cddis2016396x6.pdf]

Figure S6

A

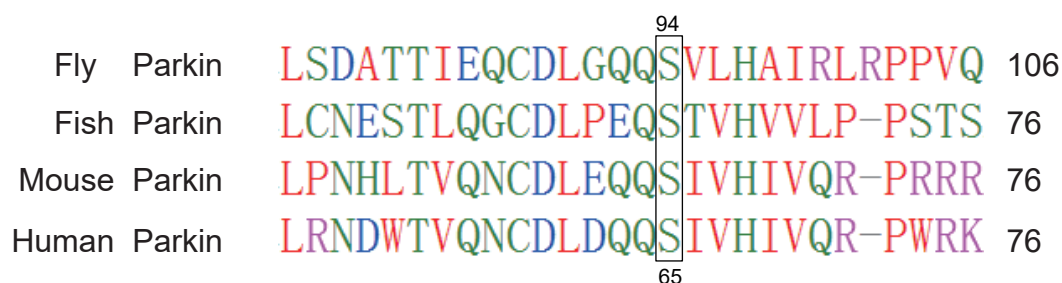

B

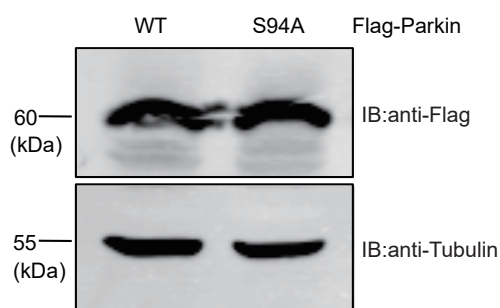

C

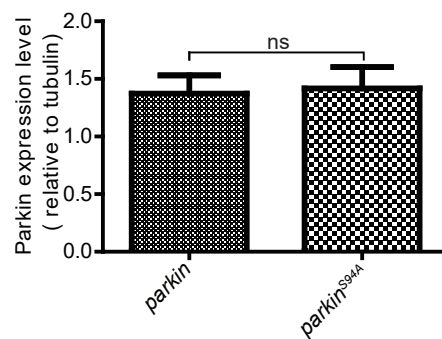

Supplement: Supplementary Figure S6 [file cddis2016396x7.pdf]

Figure S7

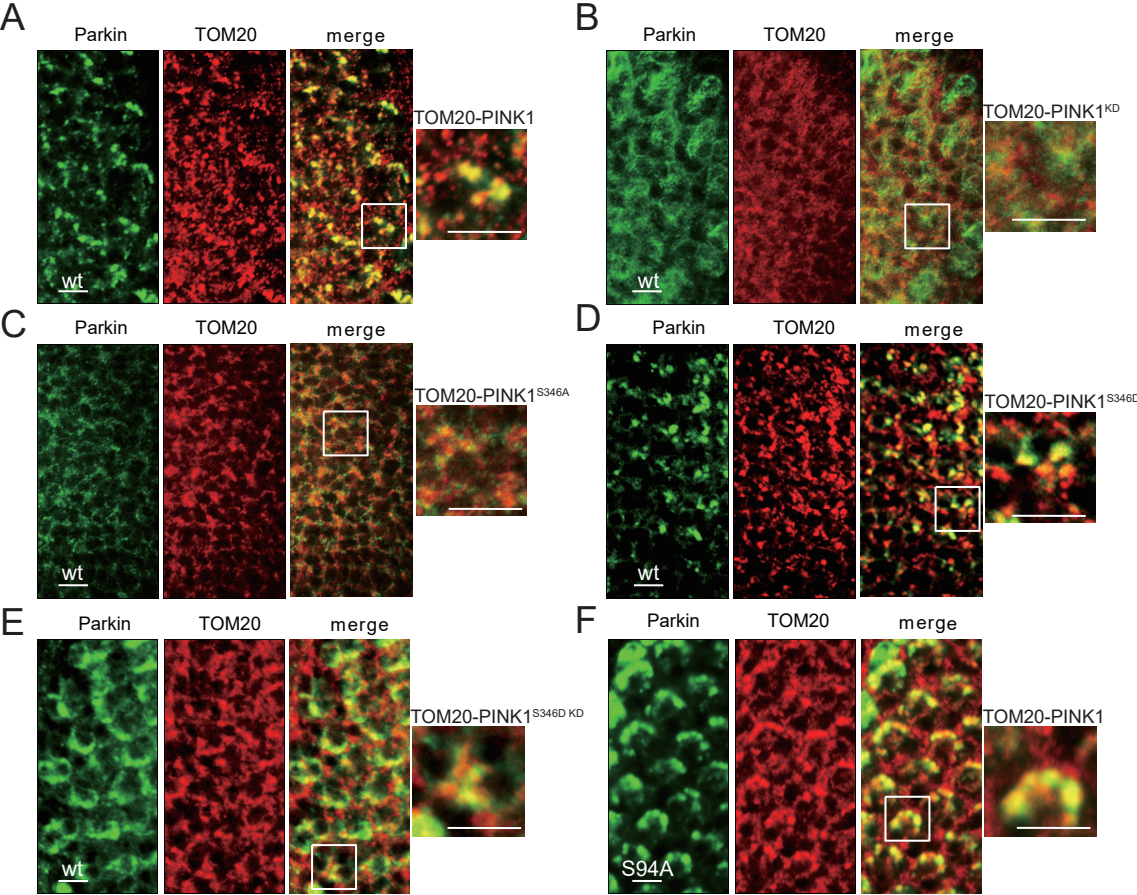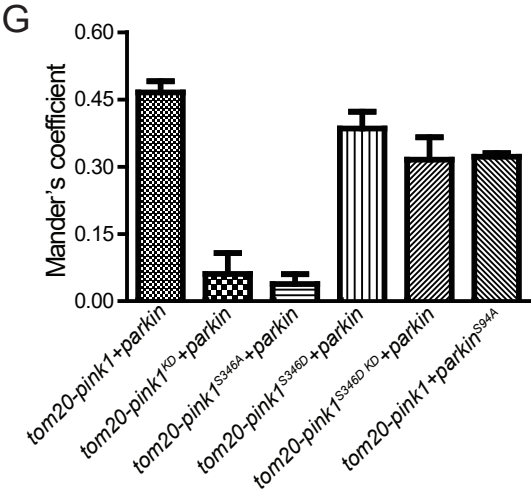

Supplement: Supplementary Figure S7 [file cddis2016396x8.pdf]

Figure S8

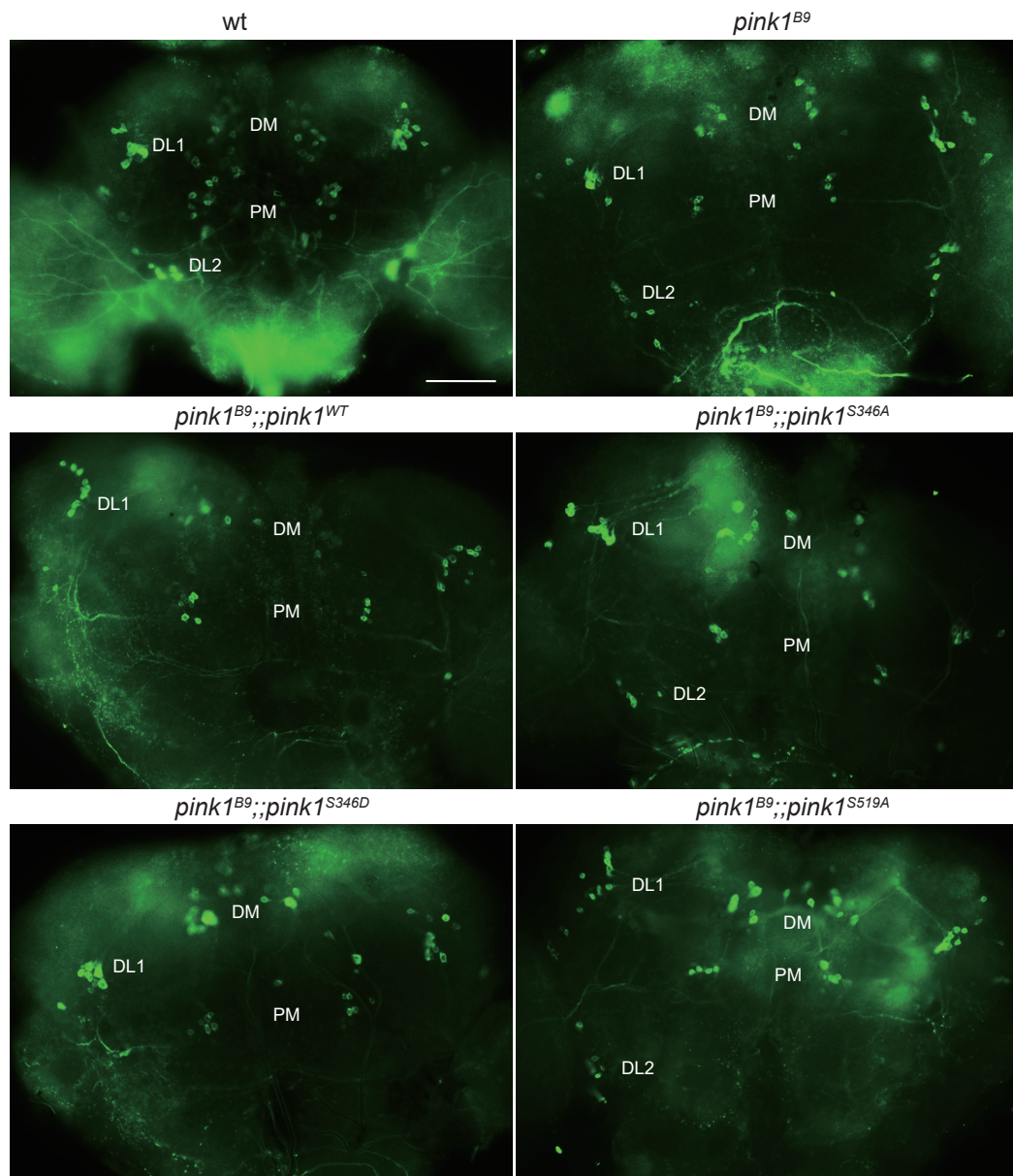

Supplement: Supplementary Figure S8 [file cddis2016396x9.pdf]
