# Supplementary material for: PINK1-dependent phosphorylation of PINK1 and Parkin is essential for mitochondrial quality control
Source: Cell Death Dis. 2016 Dec 1;7(12):e2501–. doi: 10.1038/cddis.2016.396 (PMC5261015; doi:10.1038/cddis.2016.396)
Supplement: Supplementary Figure S4 [file cddis2016396x5.pdf]

Figure S4

```
                            519
Fly    PINK1  LADKVHGLRLPYVSHDVDKGGNAALMAPE  534
Fish   PINK1  LAED-SGLKLPFSSWWVNRGGNSCLMAPE  404
Mouse  PINK1  LADQHVGLRLPFNSSSVERGGNGSLMAPE  416
Human  PINK1  LADESIGLQLPFSSWYVDRGGNGCLMAPE  417
                            402
```
